# A Chemical Study of Nasal Syphilis*Read before the Atlanta Society of Medicine, Nov. 17, 1896.

**Published:** 1897-01

**Authors:** Dunbar Roy

**Affiliations:** Atlanta, Ga.; Professor Opthalmology and Otology in Southern Medical College, Oculist and Aurist t Southern Railway, Home for the Friendless, etc.


### Southern Medical Record.

A MONTHLY JOURNAL OF MEDICINE AND SURGERY.

VOL. XXVII. ATLANTA, GA., JANUARY, 1897. No. 1

### Original Articles.

### A CLINICAL STUDY OF NASAL SYPHILIS.*

BY DUNBAR ROY, A. B., M. D., ATLANTA, GA.,

Professor Opthalmology and Otology in Southern Medical College, Oculist and Aurist t Southern Railway, Home for the Friendless, etc.

No disease has probably received more untiring discussion and none more varied in its manifestations than syphilis. To the specialist and to the general practitioner it is likewise as much of importance as it is to the syphilographer. It is one disease, like tuberculosis, which is pre-eminently general to the whole human frame.

The object, however, of this paper to-night is not the discussion of the disease syphilis in the abstract, but the discussion of its manifestations as it occurs in the nasal cavities, and at the same time to elucidate some of my remarks by the report of clinical cases.

Let me say in the outset that I have been induced to write this article from the fact that it is a subject which has been given too little prominence and too little discussed before the general practitioner, and in the second place because cases have come under my observation which have been wrongly diag-

*Read before the Atlanta Society of Medicine, Nov. 17, 1896.

nosed even by men of recognized ability in this special iine of work. I do not mean by this that the disease syphilis has not been frequently discussed, but I mean that portion of it which relates to the nose.

It is a pity, but nevertheless true, that the profession of medicine is being overcrowded, and often with men who are proving a loss to some other avocation in life. The great ease with which medical diplomas are obtained, and at the same time without the least personal fitness for the possession of the same, will eventually bring the medical profession to the recognition of the truth of the Darwinian theory, "the survival of the fittest."

In no department of medicine is there so much quackery, or lack of skill and judgment displayed as is done by many of those who affix to their names the title of nose and throat specialist. On the other hand, in no department does it take more skill to make an accurate diagnosis and apply the appropriate treatment than it does in nasal and throat diseases, and yet, because these are hidden cavities from the public gaze, the so-called specialist may irreligiously manipulate without fear of detection. A physician opens his office with a spraying apparatus, a galvano-cautery, and a couple of nasal saws, and then he is a full-fledged specialist, ready to diagnose and treat nose and throat diseases in the most approved manner. Unfortunately, nose and throat work is too much "putty work" for the average specialist, who often saws out an exostosis or enchondroma, or cauterizes the turbinates, leaving in both instances a cicatricial scar, because, forsooth, something radical must be done in order to make the laborer more worthy of his hire. Many a nose and throat has thus been sacrificed at the altar of an over-zealous specialist. You must excuse me, gentlemen, for thus digressing from the subject-matter of this paper, but since it is a subject that requires much skill in its diagnosis and proper treatment, my main object for this digression was to show how necessary it is for the general practitioner, as well as the specialist, to be clinically familiar with the various forms of nasal disease.

In treating the subject this evening, I will adhere to the old classification of primary, secondary, and tertiary syphilis, recognizing however, the fact that in only the required form can the primary lesion appear.

*Primary* syphilis of the nose, according to all clinical observers, is extremely rare. Fournier, of Paris, in the reports from the St. Louis Hospital, has only observed five cases of chancre of the nose during his long and wonderfully rich experience. In the study of the subject O. Seifert has collected twenty-seven cases of chancre on or in the nose. According to this observer, the mode of infection was, in the large majority of cases, by means of the finger nail, thus agreeing with other authors, and the points of location in the majority of cases on the *alæ nasi*, and less frequently on the mucous membrane. Le Bart is the latest author who has made an extended study of the subject and found up to that time only thirty-seven reported cases, to which must be added four other cases reported during last year. The diagnosis of such primary lesions must be made with caution, for the signs often are by no means well defined, as was shown in a case reported by Thibierge, where the diagnosis was difficult, "owing to considerable inflammation of the surrounding parts of the nose, which inflammation was the result of his occupation (woolens) and the employment of wrong medicimentous dressings." One is very apt to err in making a diagnosis, and should by no means be too quick in his conclusions. An abrasion just at the base of the *introitus naris* is liable to become infected from various other causes besides syphilis, hence the need of caution in such cases. If such a suspected abrasion be found, the simultaneous enlargement of the submaxillary and sublingual glands will render valuable aid in making a correct diagnosis.

Thus far it has not been my good fortune to see such a case in my own practice, although I have had several where my suspicions could not be clinically verified.

*Secondary* manifestations, as well as tertiary, occur in both the acquired and hereditary form.

The secondary forms manifest themselves, according to Pierce of Chicago, into three varieties: (*a*) erythema, (*b*) condylomata, (*c*) ulceration, which latter I consider but a transition stage between the so-called secondary and tertiary syphilis.

In the beginning of the secondary form a hyperæmic or erythematous condition of the nasal mucous membrane frequently manifests itself, and is sometimes equal to an acute coryza. Such an erythematous condition differs from an acute coryza in being more gradual in its development.

Tissier has examined the nasal fossæ in twenty-five women presenting secondary cutaneous or mucous lesions. In seventeen of these he found the membrane markedly involved, seven cases presenting erythematous rhinitis, and eight syphilitic erosions. Two presented adhesions between septum and turbinates. This latter condition cannot alone be considered as indicative of syphilis, for during the last three months I have seen three cases of these adhesions, due to bad surgery on the part of specialists in ill-advised use of the electro-cautery.

In early infancy, rhinitis, or "snuffles," as it is commonly called, is frequently one of the first signs of inherited syphilis, remembering however, at the same time, that it also occurs in scrofulous or tubercular subjects. Even more, this same condition will sometimes occur in infants who have no inherited blood or lymphatic dyscrasia; hence, one is not justified in pronouncing "inherited syphilis," simply because the infant is afflicted with the "snuffles."

Such a state of affairs in infants is always unfortunate, for it not only retards the proper development of the lungs, but it is an obstacle to good nursing, since such an infant, during the act of suckling, has not the aid of unimpeded nasal respiration. In infants the nasal cavities are so small, and so much of the cavity is still in the embryonic state, that even the slightest swelling or accumulation will cause the greatest inconvenience.

Schmiegelow has called attention to a case where such dyspnœa was occasioned by the syphilitic coryza in an infant that tracheotomy had to be done.

In the next or condylomatous variety, such a condition is far less frequent than occurs on other mucous surfaces, notably the mouth and fauces, and when it does occur is so quickly followed by superficial ulceration as to scarcely be recognized in that stage.

Such a condition, however, is more frequent than is supposed, and its cause often overlooked by the experienced rhinologist. Such a case was seen by me just a year ago:

Mrs. ——, age 32, a most elegant and refined lady, consulted me in regard to a continual stopping up and discharge from her nose, which she said had been going on for some time and produced the sensation as if she had a severe cold continuously in her head. She remarked to me at the time that she did not come for treatment, but only wished to have my opinion as to whether or not the condition was curable, as she was then under the treatment of a specialist, but as she had not improved had decided to consult some one else without the knowledge of her family physician or husband.

Upon examining the interior of the nares, I found the right side mainly affected, which consisted in a superficial ulceration, œdema and polypoid degeneration of the mucous membrane of the whole length of the inferior turbinate. To my mind the specific origin of the nasal condition was evident but not wishing her to know the character of the trouble, I advised her to report her visit to her husband and let him call to see me. This he did the same day, when I told him candidly my opinion of the case, after which he admitted the fact of having had syphilis. I then told him that the only thing then needed was to impart the knowledge of the fact to the specialist then treating his wife and I was sure that she would begin to improve. This he did, and I afterwards learned that his wife improved rapidly. This was a case where, if the diagnosis had been properly made in the beginning, the treatment would have been far more efficacious and the patient thus relieved of a distressing condition. These condylomata, according to the majority of observers, are most frequent on the mucous membrane covering the inferior turbinates, and, according to my observation, at that portion which is nearest to the septum, which, in the large majority of cases is either at the posterior or anterior extremity. The clinical symptoms of this condition cannot be better described than by narrating the history of a case recently under my charge.

Mrs. S., married, age 28, consulted me on account of the continual stopping up of the right side of the nose, with all the symptoms of a severe cold in the head. Upon examination I found the mucous membrane of that side congested and œdematous, with a good deal of watery secretion, but nothing further abnormal. As is my rule in all such cases, I placed a wad of cotton saturated with a cocaine solution well into the nose, thus causing a contraction of the parts and the consequent enlargement of the nasal passage. Let me say here that I never spray cocaine in the nose, for I consider it an unnecessary procedure and frequently discomforting in its results. In this case there was found, just at the posterior extremity of the inferior turbinate, a linear ulceration of the mucous membrane covered over with a gelatinous secretion. These superficial ulcerations I find, resemble very much an area that has been cauterized with chromic acid. The patient was closely interrogated, but gave no history of syphilis. However, there was some enlargement of the submaxillary and post-cervical glands, so she was placed upon energetic anti-syphilitic remedies, with alkaline and vaseline sprays to the nose, with the result that in three weeks the nasal chamber appeared in a normal condition.

In this case there probably first existed a small condyloma, which was followed by a breaking down and superficial necrosis of the mucous membrane. The ulceration at this stage was fortunately limited to the mucous membrane, but had the diagnosis not been correctly made the whole train of symptoms, ostitis and bony necrosis, would probably have followed and a decided picture of the tertiary stage have been the result.

This case illustrates the necessity for a thorough examination at all times, if the conscientious rhinologist wishes to treat intelligently the cases which come under his professional care.

Another case illustrating the same lesion, in which the symptoms and signs were somewhat different, came under my care some ten months ago.

Mr. D., age 32, traveling man, consulted me on account of the disagreeable discharge from the right side of his nose and an uncomfortable feeling of "stuffiness." He told me in the beginning that he had been treated for a number of years for syphilis. His throat showed all the signs of old ulceration, destruction partial of the soft palate, and cicatricial bands of adhesions around the tonsils. In his nose I found a large perforation of the septum, and along the middle of the inferior turbinate of the right side a large necrotic ulcer, with the attendant œdema of the mucous membrane and profuse discharge of a muco-purulent type. In this case, the diagnosis was easily made, both from the history given by the patient and the objective signs of previous lesions.

The patient was careless in carrying out instructions, getting better, ceasing treatment, and finally passing from under my observation.

In the third stage of this affection lesions are far more commonly recognized, because of the greater destruction they produce in the nasal cavities, and consequent marked deformities which sometimes occur. This stage is called by Pierce the granulomatous, because of the granuloma formation and the consequent breaking down of the periosteum and bone substance proper. If the granuloma or superficial ulceration is not checked in its incipiency the consequent involvement of the bone is sure to follow.

Such granulomatous degeneration may occur either upon the turbinates, upon the septum, or in a case seen by me upon the floor of nasal cavity. If the mucous membrane covering the turbinates should be in contact with the septum, and ulceration should occur at that point, then a necrosis of the underlying bone is almost sure to follow. All of you, no doubt, have seen the fearful ravages and the consequent deformities made upon the nose through the action of the syphilitic virus—the destruction of the septum and the consequent falling in of the bridge, the destruction of all the tissue in the nose and the consequent fetid discharge, and even in some cases the utter destruction of all the exterior portion of the nose, leaving only two elliptical openings in the face.

A perforation of the septum does not always mean that it is syphilitic. When I first began the practice of rhinology, I was of the opinion that every perforation which was seen was syphilitic, but soon ascertained my error. It is by no means infrequent for the rhinologist to find perforations of the cartilaginous portion of the septum caused from perichondritis, the result of trauma, chemicals, etc., especially those the result of erosions, due to habitual nose-bleed, and the consequent vascular changes.

A chondritis always follows the removal of an enchondroma, and is frequently the cause of a perforation at this point. But we may put it down as a rule that all perforations of the bony septum are due to syphilis, for Schroetler and O. Seifert have shown that a syphilitic ulceration confined exclusively to the cartilaginous portion of the septum is extremely rare. Furthermore, a perforation, with the attendant ulceration around its periphery, due to syphilis, is far more unhealthy looking than those due to other causes.

I will take the liberty of giving here the history of a case in which the specific character had not been recognized by former specialists who had treated the case, and consequently received very little result for their treatment.

Miss N., age 32, upon the advice of her family physician, consulted me, late one afternoon, in regard to the best surgeon in New York to whom she might go and have a portion of her upper jaw removed for the intense pain she had been suffering for a month or more. Before making an examination she told me that she had been treated for the last year by men of acknowledged ability, who told her that she had a chronic disease of the antrum, and was so treated.

She had suffered with such intense pain for the last two months as to be compelled to resort to morphine every night in order to procure any rest whatever. At her request, I then made an examination, and found the following condition:

The right side was entirely occluded from œdema of the mucous membrane, mainly over the inferior turbinate. The patient could force scarcely any air whatever through the right side by the most strenuous exertion. Under cocaine the parts were contracted as much as possible, and by means of illumination and the probe the condition of affairs was still further studied. A necrosed condition of the septum, about the size of a dime, was found situated towards the posterior portion of the septum, an ulcer on the floor of the cavity, an ulcerated condition of the posterior portion of the inferior and middle turbinate, with consequent bony necrosis, even of the wall separating this cavity from the antrum. The patient belonged to that class where an abundant opportunity was offered for infection, although she declared never to have been infected that she knew of.

The diagnosis in this case was easy, and the steps in the treatment plainly evident. A few necrosed spinulæ of bone were removed from the turbinates and a portion of the wall separating the nasal cavity and antrum, so that a large direct communication was made between the two. The patient was instructed to douche her nose with warm carbolized water three times daily, and in addition she was placed upon increasing doses of K. I. By degrees all the necrostic tissue was removed from the interior of the nasal cavity, the process of disintegration was checked, and, after months of treatment, the patient was thoroughly restored, with the exception, however, of the loss of tissue inside and a slight tendency to catarrhal symptoms.

One of the remarkable features of this case was the fact that the intense pain left immediately after the first treatment, and never after was it necessary to resort to a narcotic.

I cannot be too urgent in insisting that a thorough examination be made of every nasal case, no matter how slight may be the symptoms, for a correct diagnosis, after all, is the sum total of the proper treatment of nasal diseases.

While it is exceptional for any case of nasal syphilis to result fatally, yet resulting deformities may often prove bad advertisements for a physician's success.

I say that such a condition is rarely fatal, yet Fournier, according to Pierce, has called attention to the manner in which syphilis may prove fatal. In such cases the ethmoid or sphenoid are involved. "Secondarily, the results may be (1) meningitis, in its various forms; (2) thrombosis of the sinuses, especially the sinus cavernosus, which lies close to the sella tursica; (3) thrombosis of the vera ophthalmia, which empties into the sinus; (4) the escape of pus, or extension of inflammation into neighboring parts, as the orbit, etc.; (5) implicating the optic or olfactory nerves, as the third, fourth and sixth pairs; (6) encephalitis circumscripta or diffusa, with abscess." The deformities which sometimes follow nasal syphilis may be both internal or external, and usually the former, since proper treatment will, in the majority of cases, arrest the disease before the exterior portions have become much involved. The turbinates may be destroyed and even the walls of the antrum, so that the nasal cavity presents one large communicating opening, as in a case reported by Dr. John Dunn. The septum may break down at any point and result in the deformity known as "saddle-shaped" nose. With such conditions and in few exceptions, there is the accompanying fetid discharge.

The probe cannot be over-estimated in its value for aiding in a correct diagnosis of nasal diseases. The touching of soft spots will sometimes reveal necrosed bone entirely unsuspected. Of especial aid is a small piece of cotton wrapped on the end of an applicator, passed over suspected necrosis, which by catching roughened surfaces of bone will expose often this pathological condition where other means have failed.

The treatment of this pathological condition must necessarily be divided into the *constitutional* and *local*, and of the two the former is far more important than the latter.

It is unnecessary for me here to burden you with the constitutional treatment of syphilis, taking it for granted that your own clinical experience is the best teacher. My own experience has been that mercury in nasal syphilis is far more beneficial than any other remedy unless decided osteitis and the consequent necrosis has appeared. Every syphilitic patient should first have a thorough mercurial course of treatment before the administration of iodides, even if it be the late tertiary forms. In some cases where the mercury can not be borne, then we must resort to the iodides, and especially the latter whenever the lesions have invaded the bony framework. Above all, we should recognize the value of the constitutional treatment.

Locally, the whole treatment may be summed up in the word, *cleanliness*. In infants the question of cleansing the nasal cavities is a very perplexing one, and yet one which should be considered far more frequently than it is, even if the condition is not specific. Babies afflicted with "snuffles," hard dry secretions filling the nasal cavities, profuse discharge, etc., are always conditions which shonld receive attention. Nursing under such conditions is often impeded. Before the child has reached the age when it may be taught to blow its own nose, then this act must be performed for it. The best method, when the secretion is not hard and dry, is to remove the same by means of the air douche, applied to one nostril while the other remains open. If the child cries, so much the better, for the post-nasal space is then cut off.

This should be repeated at intervals during the day, and if the nasal secretion is dry and hard a few drops of benzoinated albolene should be poured into the cavities to soften the mass before an attempted expulsion. If the discharge should be offensive in odor, we may have either beginning atrophic catarrh or syphilitic rhinitis. Other signs will aid us in the differential diagnosis, but in any case the nasal cavities must be thoroughly cleansed. After the above has been done, *i. e.*, a thorough cleansing as far as possible, a method recommended by Pierce may be used. This consists in passing through the nose, by means of a glass tube, the fumes of sublimated calomel.

In adults the cleansing process can be much more thoroughly executed. Various cleansing solutions can be used, but I have found none which answers more satisfactorily than a solution of bicarbonate of soda, to which a couple of drops of carbolic acid have been added. These solutions are preferably used by means of the douche, if the nasal chambers are thoroughly patulous, otherwise it is best to pour the solution into the nares by means of a medicine dropper or the finger douche, the patient saying "Ah!" at the same time, so as to close the post-nasal space, and the nose being thoroughly blown immediately afterwards. At the office the physician can, of course, with sprays and cotton applicators thoroughly cleanse the nasal chambers. For ulcerations I have found nothing better than nitrate of silver solutions, and in severe cases even the solid stick. When sequestræ of necrosed bone exist they must be removed before complete reparation will commence. In conclusion, let me say that nasal syphilis is far more frequent than is usually supposed, therefore a thorough history should be had of every case.

